# A brief review on the mechanisms and approaches of silk spinning-inspired biofabrication

**DOI:** 10.3389/fbioe.2023.1252499

**Published:** 2023-09-06

**Authors:** Xuan Mu, Reza Amouzandeh, Hannah Vogts, Elise Luallen, Milad Arzani

**Affiliations:** Roy J. Carver Department of Biomedical Engineering, College of Engineering, University of Iowa, Iowa City, IA, United States

**Keywords:** additive manufacturing, Hofmeister effects, silk fibroin, sustainability, tissue scaffolds

## Abstract

Silk spinning, observed in spiders and insects, exhibits a remarkable biological source of inspiration for advanced polymer fabrications. Because of the systems design, silk spinning represents a holistic and circular approach to sustainable polymer fabrication, characterized by renewable resources, ambient and aqueous processing conditions, and fully recyclable “wastes.” Also, silk spinning results in structures that are characterized by the combination of monolithic proteinaceous composition and mechanical strength, as well as demonstrate tunable degradation profiles and minimal immunogenicity, thus making it a viable alternative to most synthetic polymers for the development of advanced biomedical devices. However, the fundamental mechanisms of silk spinning remain incompletely understood, thus impeding the efforts to harness the advantageous properties of silk spinning. Here, we present a concise and timely review of several essential features of silk spinning, including the molecular designs of silk proteins and the solvent cues along the spinning apparatus. The solvent cues, including salt ions, pH, and water content, are suggested to direct the hierarchical assembly of silk proteins and thus play a central role in silk spinning. We also discuss several hypotheses on the roles of solvent cues to provide a relatively comprehensive analysis and to identify the current knowledge gap. We then review the state-of-the-art bioinspired fabrications with silk proteins, including fiber spinning and additive approaches/three-dimensional (3D) printing. An emphasis throughout the article is placed on the universal characteristics of silk spinning developed through millions of years of individual evolution pathways in spiders and silkworms. This review serves as a stepping stone for future research endeavors, facilitating the *in vitro* recapitulation of silk spinning and advancing the field of bioinspired polymer fabrication.

## 1 Introduction

**FIGURE 1:**
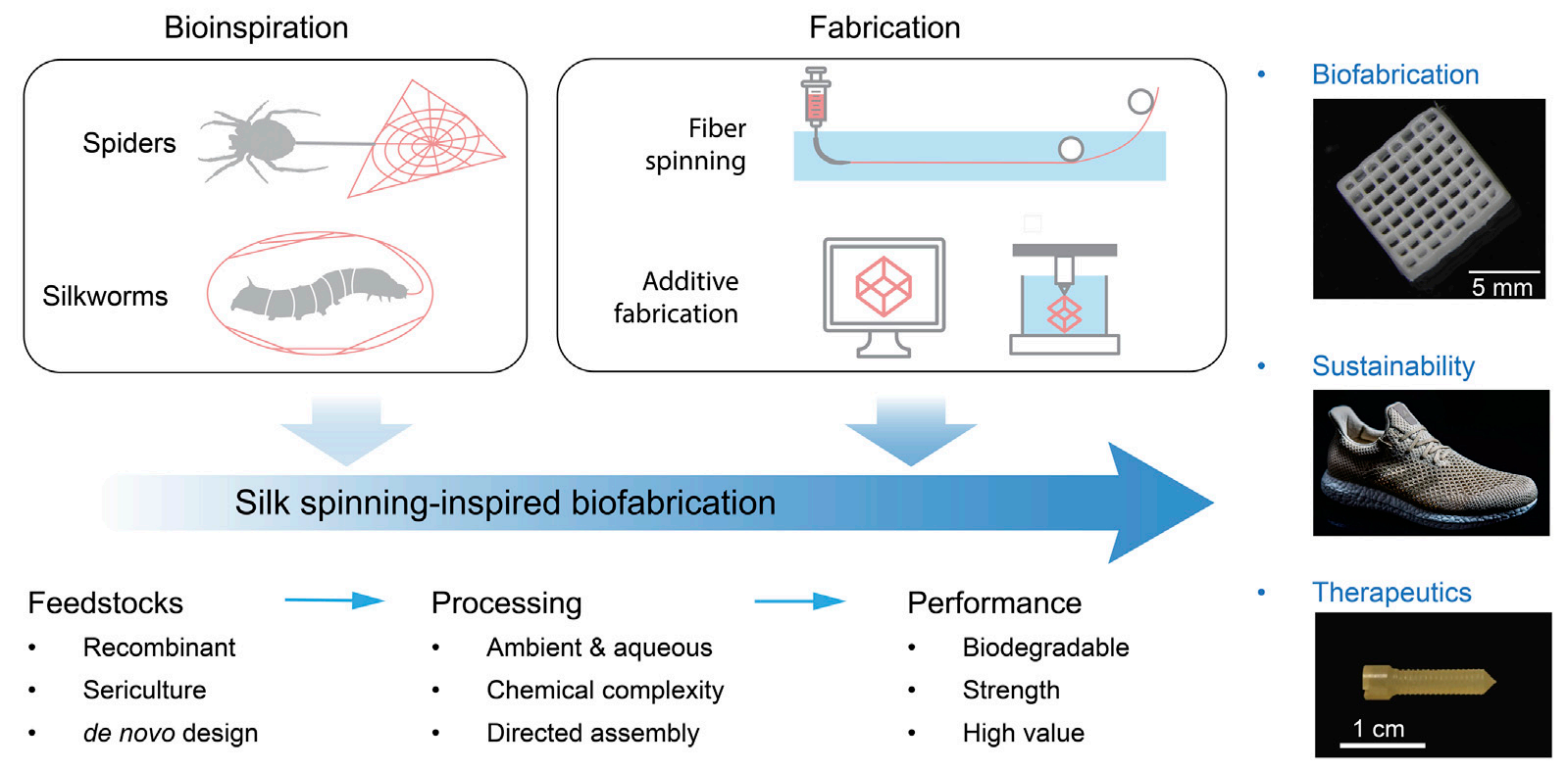
Schematic of silk spinning-inspired biofabrication promising to promote advanced manufacturing, sustainability, and clinical therapeutics. The exemplary silk structures include three-dimensional (3D)-printed lattices, plastics-free athletic footwear, and bone screws. Reproduced with permission from Springer Nature (Guo et al., 2020b) and the American Association for the Advancement of Science (Service, 2017).

Silk spinning is a sophisticated fabrication process turning aqueous proteinaceous feedstocks into mechanically exceptional materials and structures, which represents an engineering marvel developed by millions of years of natural evolution (Magoshi et al., 1996; Vollrath and Knight, 2001; Rising and Johansson, 2015). Of note, silk spinning seems fundamentally different from modern industrial manufacturing of synthetic polymers and plastics that is based on fossil-derived feedstocks, intense energy input, and accumulated environmental pollution. The characteristics of silk spinning are most likely in three essential aspects, including the feedstocks, processing conditions, and product performance (Figure 1).

The feedstocks of silk spinning are the aqueous solutions of concentrated silk proteins (25–50 wt%) (Hijirida et al., 1996; Laity et al., 2015). Silk proteins are not a component of the extracellular matrix (ECM), but it has the same chemical building blocks, amino acids, as the living systems and usually exhibits controlled degradation and negligible immunogenicity (Wang et al., 2008; Deptuch et al., 2022). A monolithic proteinaceous structural basis is expected to enable cell-mediated matrix remodeling and to promote host-implant integration and biocompatibility, which is instrumental to a magnitude of bioengineered tissue scaffolds (Torculas et al., 2016; Holland et al., 2019; Guo et al., 2021).

The processing conditions of silk spinning are fully aqueous and ambient, which is energy-saving and environment-friendly but also helps keep the biofunctions of integrated molecules that may be lost due to heating or organic solvents. This feature is highly desired for manipulating the cellular microenvironment (Zhang et al., 2020) and devising drug delivery systems (Wenk et al., 2011). During the spinning process, the silk proteins are directed to assemble into hierarchical structures, from secondary structures, micelles, nanofibrils, and granules to fibers (Ebrahimi et al., 2015; Ling et al., 2018; Lin et al., 2019; Su et al., 2020). Silk nanofibers are a versatile tool for making valuable materials (Dong et al., 2016; Ling et al., 2016; Müller et al., 2020). In addition, silk spinning is usually carried out at around tens of milliliters per second (Shao and Vollrath, 2002), which is faster than the month-long growth of other structural proteins, such as tendon and mussel byssus, thus promising for scaling up manufacturing productivity. The interactions between silk proteins and the information-rich solvent environment may represent an avenue for devising fabrication conditions (Mu et al., 2020a).

Silks, especially spider dragline silks, are renowned for their superior mechanical performance, partly resulting from the orchestrated organization of polypeptide chains. For example, dragline silk is as strong as high-tensile steel (1.1 GPa vs. 1.5 GPa) but is lighter in weight by roughly six times (1.3 kg/m^3^ vs. 7.8 kg/m^3^) (Omenetto and Kaplan, 2010). The structure-property relationships or the structural basis of the mechanical performance of silk materials have been extensively investigated (Giesa et al., 2011; Tokareva et al., 2014), which underpins the further investigation of the effect of processing conditions or the structure-process-property relationship of silk spinning.

Silk spinning, due to its exceptional manufacturing merits, has been recognized as an important source of inspiration for the development of advanced biofabrications, such as footwear and biomedical devices (Figure 1). The athletic apparel and footwear company, Adidas, has worked with AMSilk to introduce the first performance shoe made in 100% artificial silk fibers (Service, 2017). The shoes are characterized by 15% lighter in weight, full biodegradability, and the absence of plastics. In addition, silk proteins have been processed via solvent-based (Perrone et al., 2014) or thermoplastic molding (Guo et al., 2020b) into bone screws, a common implantable device to assist in the healing of bone defects. The silk-based bone screw exhibited benefits compared to synthetic polymers and metals, including ease of implantation, biodegradability, and minimal inflammatory response.

The manufacturing features of silk spinning would be generally valuable to help address the emerging challenges in sustainability and healthcare. This article will briefly review essential aspects of silk spinning mechanisms and the state-of-the-art bioinspired approaches. We will discuss universal/cross-species features, not species-specific, of silk spinning, as spiders and silkworms share a substantial set of mechanisms despite their separate evolution pathways (Andersson et al., 2016). For insect silks, the focus will be placed on silk fibroin from the *Bombyx mori* (*B. mori*) silkworms; for spider silks, the name of a certain spider will be provided when necessary. Furthermore, a concise section is dedicated to the promise of silk spinning as a holistic approach to promoting material, energy, and environmental sustainability. We envision this brief review to stimulate further efforts in devising silk-spinning-inspired biofabrication.

## 2 Mechanisms of silk spinning

Despite substantial progresses in the past decades (Vollrath and Knight, 2001; Omenetto and Kaplan, 2010; Eisoldt et al., 2011; Liu Y. et al., 2019; Guo et al., 2020a; Mu et al., 2020a), the understanding of silk spinning remains incomplete and may be promoted by embracing recently evolved concepts and techniques (Rising and Johansson, 2015; Moreno-Tortolero et al., 2023). The fundamental mechanisms of silk spinning are tightly related to the directed assembly of silk proteins across hierarchical length scales, which is characterized by a phase transition from liquid to solid underlying the fiber spinning but also the precise manipulation and hierarchical organization of silk proteins that lead to the superior mechanical performance of bulk silk materials. It has been suggested that the solvent cues in the native spinning apparatus, i.e., the aqueous solution of silk feedstock, roughly including pH, salt ions, and water content, may direct the assembly of silk proteins in the absence of external heating and extensive energy input (Magoshi et al., 1996; Heim et al., 2009; Andersson et al., 2016). The interactions between the solvent cues and the silk proteins thus seem to be a primary molecular basis for the fiber spinning at the macroscopic level. In the following sections, we will discuss two essential components in the complicated mechanisms of silk spinning, the molecular design of silk proteins and the solvent cues along the spinning apparatus.

### 2.1 Molecular designs of silk proteins

**FIGURE 2:**
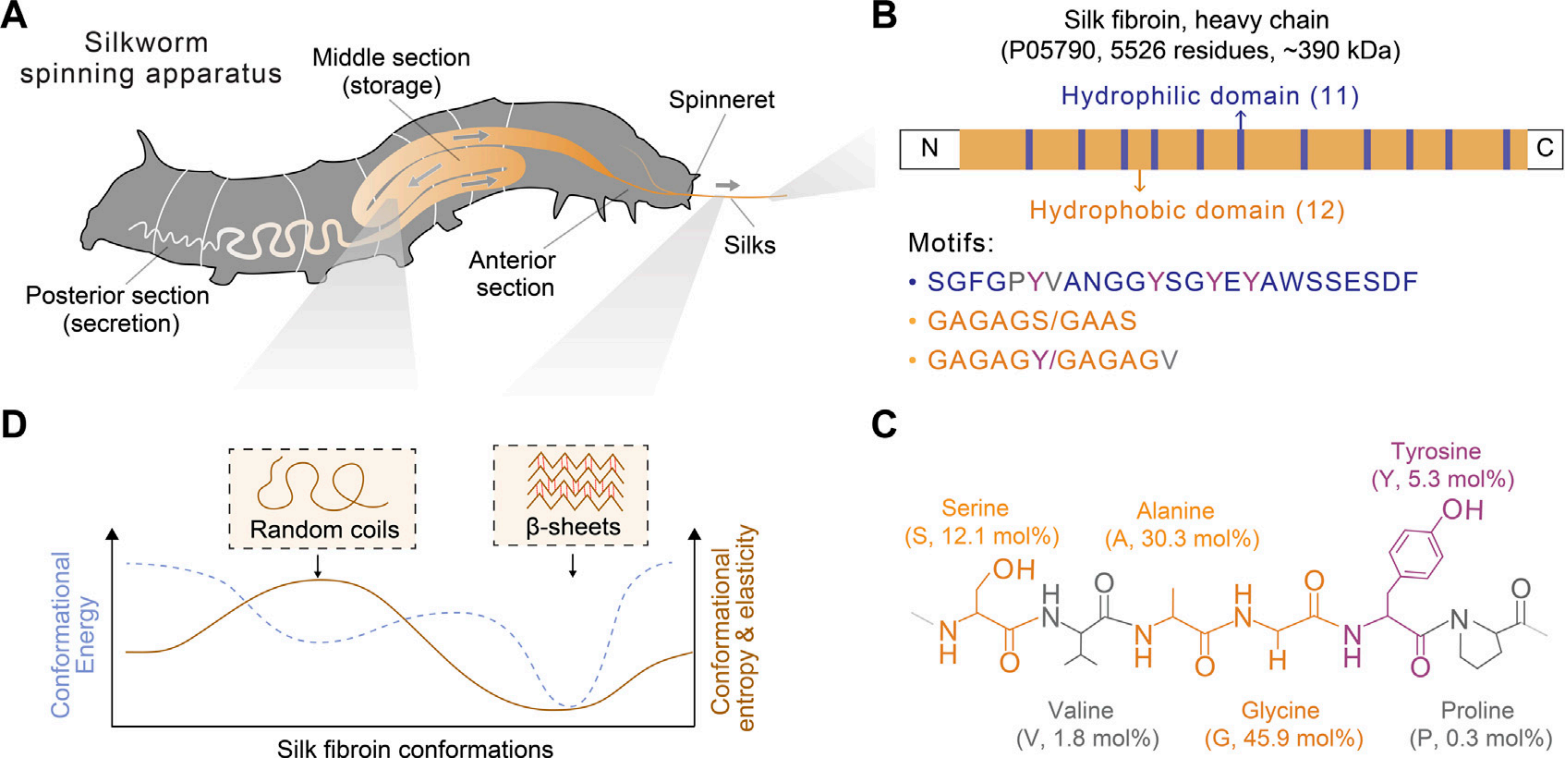
Schematics of the spinning apparatus and silk fibroin proteins of *B. mori* silkworms. **(A)** Spinning apparatus of *B. mori* silkworms. **(B,C)** Molecular design of silk fibroin heavy chain and the structural and composition of primary amino acids. The molar percentage is also indicated in parentheses. **(D)** Conformational polymorphism of silk fibroin. Two conformations, random coils and β-sheets, are assumed to adopt distinct energy and entropy.

Silk proteins are diverse across multiple species, such as spiders and insects, partly due to the separated evolution pathways and distinct habitats (Craig, 1997; Gatesy et al., 2001; Arakawa et al., 2022). Despite their diversity, various silk proteins exhibit certain highly conserved features in the molecular design (Figure 2), for example, alternating hydrophilic and hydrophobic domains, the abundance of certain amino acids, motifs, polymorphic conformations, and the formation of higher-level structures, i.e., β-sheets, micelles, and nanofibers. These universal features seem important to the spinning process and may represent a general scientific framework for the rational design of silk spinning-mimetic fabrication. In particular, the molecular understanding of the designing principles has inspired the development of high-performance synthetic polymers (Wu et al., 2017; Dou et al., 2019; Mohammadi et al., 2019; Shi et al., 2023). Furthermore, the recombinant DNA technology and the advances in protein engineering (Koga et al., 2012; Huang et al., 2016) underpin the creative modulation of amino acid sequences to give rise to *de novo*, genetically modified, and chimeric silk proteins, which may bring benefits in improving production yield (Tucker et al., 2014; Decker, 2018), extending functions (Gomes et al., 2011; D'Amone et al., 2023), and facilitating fiber spinning (Teulé et al., 2009; Andersson et al., 2017; Saric et al., 2021).

Below, we provided a brief discussion on the molecular design of silk proteins in three aspects, amino acid composition, motif, and conformation. Both spider and silkworm silks are similar in the abundance of certain amino acids, such as glycine (G), alanine (A), and proline (P) (Zhou et al., 2001; Rauscher et al., 2006). For example, the heavy chain of *B. mori* silk fibroin contains 45.9 mol% glycine, 30.3 mol% alanine, and 0.3 mol% proline (Figure 2; Table 1) (Murphy and Kaplan, 2009); one component of spider dragline silks, major ampullate spidroin 1 (MaSp1), contains around 42.3 mol% glycine, 32.7 mol% alanine, and 0.4 mol% proline (Ayoub et al., 2007; Malay et al., 2022). Glycine and alanine are hydrophobic with small side chains, including a hydrogen atom and a methyl group. From the perspective of steric effects, the small side chains enable chain flexibility and facilitate the tight stack of polypeptide chains and the formation of β-sheets. In addition, proline is anomalous in the nitrogen-involved, five-membered ring in its backbone, which markedly restricts the angle of the polypeptide bonds and usually prevents the formation of β-sheets (Morgan and Rubenstein, 2013). Thus, silk fibroin heavy chain and the MaSp1 exhibit a tendency to form β-sheets; the dynamics of the conformational transition can be tuned by some strategies (Raia et al., 2017; Mu et al., 2022b). In comparison, other structural proteins with slimier glycine ratio yet an elevated ratio of proline, such as resilin (glycine 39–42 mol% and proline 7–12 mol%) and elastin (glycine 33 mol% and proline 12 mol%), tend to adopt random coil conformation (Rauscher and Pomès, 2012). Furthermore, silk fibroin contains around 5.3 mol% tyrosine that has a phenolic amphipathic side chain and is both hydrophobic and polar, thus offering versatile physicochemical properties (Table 1). The hydroxyl group makes tyrosine more polar than phenylalanine, which enables hydrogen bonding and improves the solubility in water; the aromatic ring primarily renders tyrosine hydrophobic and enables hydrophobic interactions. Also, the redox capability of tyrosine has been exploited to process silk proteins, including chemical modification (Sahoo et al., 2021; Liu et al., 2022), hydrogel crosslinking (Applegate et al., 2016; McGill et al., 2017; Choi et al., 2021; Mu et al., 2022b), and three-dimensional (3D) printing (Costa et al., 2017; Mu et al., 2020b).

**TABLE 1:** The composition and properties of primary amino acids in the heavy chain of silk fibroin (P05790).

Amino acid	Abbrev	Molar ratio (%)	Side chains		Hydrophobic	Polar	β-sheet motifs
Glycine	Gly G	45.9	Hydrogen	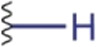	Yes	-	Yes
Alanine	Ala A	30.3	Methyl	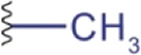	Yes	-	Yes
Proline	Pro P	0.3	Pyrrolidine ring	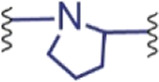	Yes	-	No
Tyrosine	Tyr Y	5.3	Phenol ring	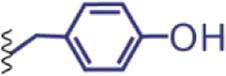	Yes	Yes	Yes
Serine	Ser S	12.1	Hydroxymethyl	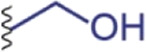	-	Yes	Yes
Valine	Val V	1.8	Isopropyl	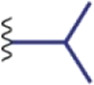	Yes	-	Yes

In addition, the incorporation of certain amino acids can manipulate the biocompatibility and mechanical properties. For example, the Arg-Gly-Asp sequence can be incorporated into silk proteins to promote cell adhesion and osteoblastic differentiation (Bini et al., 2006; Morgan et al., 2008). According to the propensity of amino acids to form β-sheets, the replacement of alanine with isoleucine has improved the mechanical performance of artificially spun silks, as discussed in Section 3.1 (Johansson et al., 2010).

Silk proteins are large proteins (larger than 300 kDa) and analogous to linear block copolymers, which contain non-repetitive N- and C- terminal domains and tens of repeated segments dominated by either hydrophobicity or hydrophilicity (Bini et al., 2004) (Figure 2B). The length of the silk fibroin heavy chain and the MaSp1 is dominated by hydrophobic motifs, such as AAAAAA (for MaSp1) and GAGAGS (for silk fibroin, S is serine). The polypeptide chains of silk protein are believed to form micelle-like structures (Jin and Kaplan, 2003; Lu et al., 2012) and liquid crystals (Vollrath and Knight, 2001). These assembled and intermediate structures are suggested to play a critical role in promoting the ambient storage of highly concentrated silk dope and mediating the fibrillogenesis in silk spinning. Textured birefringence, as optical evidence of liquid crystals, has been observed in native silk dope found in Nephila edulis (*N. edulis*) spiders (Knight and Vollrath, 1999) and *B. mori* silkworms (Asakura et al., 2007).

Silk proteins are characterized by adopting multiple functional conformations, such as random coils and β-sheets (Figure 2D). Random coils are not a single conformation but a range of rapidly interchangeable conformations, making silk proteins water-soluble and constituting semi-amorphous regions; β-sheets are pleated polypeptide chains, especially hydrophobic domains, in a sheet-like structure primarily via hydrogen bonds, rendering silk proteins water-insoluble and constituting crystalline regions (Koh et al., 2015; Oktaviani et al., 2018). The size of the β-sheet nano-crystals is related to the ultimate strength and stiffness of silks (Keten et al., 2010). Silk proteins may adopt other secondary structures, such as α-helices and β-turns, characterized by infrared (IR), circular dichroism (CD), nulcear magnetic resonance (NMR), and Raman spectrum (Rousseau et al., 2004; Hu et al., 2006; Lefevre et al., 2008). However, according to NMR, silk fibroin is highly unlikely to adopt α-helices compared to spider silks (Asakura et al., 2015; Asakura, 2021). This result is attributed to the differences in the amino acid composition of the motifs (e.g., GAGAGS vs. AAAAAA) and the lack of a good reference of α-helices for the vibrational spectrum (Asakura et al., 2015).

The conformational transition of silk proteins from random coils to β-sheets underpins the phase transition, solubility alterations, and aggregations of silk proteins and, thus, is critical to silk spinning and artificial fabrications with silk-based feedstocks. Methanol and other polyols can induce β-sheets of silk proteins and are widely used in the artificial fabrication with silk proteins, such as fiber spinning (Xia et al., 2010; Koeppel and Holland, 2017; Bowen et al., 2018) and 3D printing (Ghosh et al., 2008; Sun et al., 2012; Jose et al., 2015). Importantly, the use of organic solvents in the fabrication process introduces a disparity from the native conditions of silk spinning and represents a different mechanism. Such disparity in silk structures may compromise the control over mechanical performance and the downstream biomedical applications.

### 2.2 Spinning apparatus and solvent cues

**FIGURE 3:**
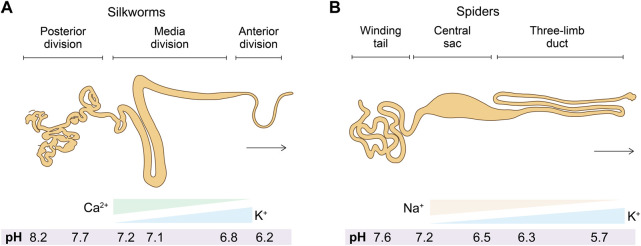
**(A,B)** Schematics of the anatomic structures and solvent cues (salt ions and pH) in the spinning apparatus of silkworms and spiders.

The spinning apparatus is a specialized tapering tubular epithelium organ that underpins the secretion, storage, transportation, and spinning of silk proteins (Knight and Vollrath, 1999; Asakura et al., 2007) (Figure 3). The spinning apparatuses found in silkworms and spiders are different in terms of, for example, the evolution of origin and the number of spinnerets. The spinning apparatus in silkworms originates from salivary glands, includes three distinct divisions (posterior, media, and anterior), and has a pair of spinning apparatus that fuse into one spinneret. The two major silk proteins of silkworms, fibroin and sericin, are secreted at the posterior and media divisions, respectively. As a result, the silkworm silk is composed of two brins (largely fibroin) conglutinated by the sericin binder or coating (Chen et al., 2012). In comparison, the spinning apparatus of spiders originates from the epidermal invaginations of the abdomen (opisthosoma) and is divided into a winding tail, central sac, and three-limb duct (Andersson et al., 2016).

The solvent environment along the spinning apparatus, including various solvent cues and flow dynamics, seems to be delicately controlled over a certain dynamic range, indicating a critical role in silk spinning. The semi-quantitative analysis of element composition revealed the spatial distribution and multiple-fold change of salt ions, including sodium (Na^+^), chloride (Cl^−^), potassium (K^+^), phosphate (PO_4_^3−^), and sulfate (SO_4_^2−^) (Knight and Vollrath, 2001; Zhou et al., 2005a). Microelectrode and pH-indicating dyes suggested a gradually lowered pH gradient along the spinning apparatus from around 8.0 to 6.0 (Foo et al., 2006; Miyake and Azuma, 2008; Andersson et al., 2014; Domigan et al., 2015). Water is also removed from the spinning dope, most likely via absorption through the epithelium, leading to an increased solid content from around 25 wt% to over 90 wt% (Vollrath et al., 1998; Kojic et al., 2004). In addition to these solvent cues, spinning speed and shear rate within the spinning apparatus have been recognized as important factors in silk spinning (Shao and Vollrath, 2002; Sparkes and Holland, 2017). Computational simulation on the effect of spinning speed on the flow behavior is promising to offer insights into the development of biomimetic spinning approaches (Breslauer et al., 2008; Kinahan et al., 2011). This section will discuss the current understanding of the three major solvent cues, including salt ions, pH, and water content, and their potential role in devising biomimetic biofabrication.

#### 2.2.1 Salt ions

The element analysis of the spinning dope along the spinning apparatus has been carried out using a range of techniques, including cryo-scanning electron microscope (SEM)-energy dispersive X-ray (EDX) (Knight and Vollrath, 2001), proton-induced X-ray emission (PIXE) (Zhou et al., 2005a), atomic adsorption spectroscopy (AAS) (Zhou et al., 2005a; Zhou et al., 2005b), inductively coupled plasma mass spectroscopy (ICP-MS) (Zhou et al., 2005a), ICP-AAS (Wang et al., 2017; Liu Q. et al., 2019), and ICP-optical emission spectroscopy (ICP-OES) (Laity et al., 2019). Most likely due to the differences in the detection mechanisms of these techniques and the sample preparation, there is no consensus on the exact salt concentration, but the overall trend of salt ions along the spinning apparatus from the posterior to the anterior and from the tail to the duct has been identified. For example, the two most abundant metal elements in the spinning apparatus of silkworms and spiders demonstrated almost the same trend K increase and Ca or Na decrease (Magoshi et al., 1996; Knight and Vollrath, 2001; Chen et al., 2004; Zhou et al., 2005a) (Figure 3). Furthermore, partly due to the unique anatomic feature of the anterior division/duct, such as the small diameter and the tough cuticular intima at the site of fiber formation (Domigan et al., 2015), the exact concentrations of salt and other solvent cues remain inaccessible (Rising and Johansson, 2015).

Salt ions have been known to affect the conformational transition, thermostability, and viscoelastic properties of silk proteins. Almost all metallic ions found in the native spinning apparatus of silkworms and spiders have been found to induce the formation of β-sheets of silk proteins, which include cupric (Cu^2+^) (Zhou et al., 2003; Zhou et al., 2005a), Ca^2+^ (Zhou et al., 2004; Koeppel et al., 2020), K^+^ (Chen et al., 2002a; Chen et al., 2002b; Dicko et al., 2004; Slotta et al., 2007; Ruan et al., 2008; Slotta et al., 2008; Lammel et al., 2010; Koeppel et al., 2020), Na^+^ (Dicko et al., 2004; Ruan and Zhou, 2008), magnesium (Mg^2+^) (Zhou et al., 2005a), zinc (Zn^2+^) (Zhou et al., 2005a), and ferric (Fe^3+^) ions (Ji et al., 2009). In other reports, under different experimental conditions (such as the composition and concentration of silks and the concentration of salts), these metallic ions have been found to exhibit different effects. For example, K^+^ and Na^+^ were found to increase the content of random coils and facilitate the breakdown of the molecular network (Zhou et al., 2005a); Ca^2+^ at a concentration higher than 10 mg per gram of silk protein inhibited the conformational transition (Zhou et al., 2004); Na^+^ was suggested to impede the conformational transition and the corresponding aggregation, thus beneficial for the storage of silk proteins (Hagn et al., 2010; Hagn et al., 2011); Ca^2+^ and Mg^2+^ were found to stabilize the predominantly disorder state of the spider silk protein (Dicko et al., 2004). Although cations have been predominately studied, the effect of anions, such as PO_4_^3−^, on silk proteins also elicited substantial research attention (Rammensee et al., 2008; Eisoldt et al., 2010; Humenik et al., 2014), such as ion-induced liquid-liquid phase separation (Malay et al., 2020).

The mechanisms regarding the effects of salt ions on silk proteins for silk spinning are yet to be fully understood. One hypothesis is based on the non-specific electrostatic interactions between positively charged metallic ions, such as Ca^2+^ and K^+^, and negatively charged groups, such as carboxyl groups of glutamate and aspartate amino acids (Kim et al., 2004; Zhou et al., 2004). Divalent Ca^2+^ may form a transient “salt bridge” to prompt chain interactions, while monovalent K^+^ may modulate this behavior by electronic shielding. On the basis of the electronic interaction-involved crosslinks, a model called “sticky reptation” (Leibler et al., 1991) is introduced to explain the quantitative effects of metallic ions on the macroscopic rheological behaviors of silk protein solutions (Koeppel et al., 2020; Schaefer et al., 2020).

Another hypothesis proposed that specific domains of silk proteins will interact with metal ions, for example, via metal coordinate bonds. The metal coordinate bonds have been found in a variety of structural proteins and play a central role in the functionality, including dope-Fe bonds in mussel byssus cuticles and phosphoserine-Ca bonds in sandcastle worm glue (Degtyar et al., 2014). The hydrophilic domain of silk fibroin, containing histidine, glutamine, and aspartate (Figure 2), has been suggested to provide binding sites for Fe^3+^ (Ji et al., 2009) and Cu^2+^ (Zhou et al., 2005a). The GYG motif in the hydrophobic domain is also suggested to bind K^+^ (Ruan et al., 2008). In particular, the GYG is highly conserved in the sequence of K^+^-selective channel proteins (Doyle et al., 1998).

The third hypothesis is based on the specific ion effects on the macroscopic aggregation of proteins in aqueous solutions, i.e., Hofmeister-type salting out (Kim et al., 2005; Heim et al., 2009). The Hofmeister series ranks the capability of salt ions to precipitate proteins, which is related to the inherent properties, composition, and concentration of salt ions via interactions with the backbone and negatively charged side chains (Kunz et al., 2004; Zhang and Cremer, 2006; Zhang and Cremer, 2009; Lo Nostro and Ninham, 2012). The ions in the Hofmeister series are divided into kosmotropes and chaotropes; the former usually exhibit a stronger capability to salt out proteins than the latter. Kosmotropic ions are weakly hydrated cations and strongly hydrated anions, such as K^+^ and SO_4_^2−^; chaotropic ones are strongly hydrated cations and weakly hydrated anions, such as Ca^2+^, Na^+^ and Cl^−^. The mechanisms of the Hofmeister salt ions on the macroscopic aggregation of other macromolecules have been investigated, including elastin-like polypeptide (Rembert et al., 2012), lysozyme (Zhang and Cremer, 2009), and poly(N-isopropyl acrylamide) (PNIPAM) (Heyda and Dzubiella, 2014). The property of kosmotropic and chaotropic ions seems in line with the gradient of ions along the spinning apparatus, where Ca^2+^ and Na^+^ contribute to the storage of silk proteins, and K^+^ facilitates the sol-gel transition. Notably, under certain solvent conditions (1 wt% silk proteins and 0.5 M salts), the conformational change and solubility of silk proteins did not follow the Hofmeister series (Dicko et al., 2004). In addition, the Hofmeister effects have been exploited to fabricate high-performance hydrogels (Jaspers et al., 2015; He et al., 2018; Wu et al., 2021), which, however, is largely based on the close packing of polymer chains rather than a hierarchical molecular assembly. Therefore, the role of the Hofmeister-type salt ion effects in silk spinning may require further investigation.

#### 2.2.2 pH

The spinning apparatus in silkworms and spiders exhibits a pH gradient that gradually decreases. In spiders, the pH is lowered from 7.6 to 5.7 (Andersson et al., 2014); the pH in silkworms is from 8.2 to 6.2 (Domigan et al., 2015) (Figure 3). Notably, the pH cannot be measured by microelectrode in the narrow part of the duct and the anterior part due to the anatomic features (Andersson et al., 2014; Domigan et al., 2015). The spatial pH gradient is generated and maintained most likely by the proton pump in the epithelium and active carbonic anhydrase (CA) (Andersson et al., 2014; Domigan et al., 2015).

The gradually acidified environment along the spinning apparatus is suggested to play an important role in silk spinning, which is to solubilize silk proteins during storage and to initiate the aggregation of silk proteins for spinning. The effects of pH on silk proteins are perhaps based on two mechanisms. The first one is the general effect of pH on the surface charge of proteins. A pH closer to the isoelectric point of silk proteins (silk fibroin, 4.4; spidroin, 4.22) (Dicko et al., 2004; Foo et al., 2006) will reduce the surface charge of the silk proteins and the electrostatic repulsive forces between polypeptide chains, thus promoting chain interactions and protein aggregation. The second one is the pH-sensitive relay of the N-terminal domain (NT) (Askarieh et al., 2010; Kronqvist et al., 2014). The NT of spidroin is conserved across species (Gaines et al., 2010), indicating the broad applicability. At pH 7.0, the NT remains monomer and facilitates the dissolution of silk proteins; at a lower pH, around 6.4, the NT forms dimers and initiates aggregation, characterized by the formation of nanofibrils and solution turbidity, in comparison to the recombinant mini-spidroin without the NT (Askarieh et al., 2010; Landreh et al., 2010). The pH-sensitive relay also seems to depend on the salt concentration (Hagn et al., 2011; Rising, 2014). The vapors of acetic acid (pH, 2.0) and ammonia (pH, 7.0) were used to treat native silk dopes from silkworms, which leads to reversible gelation, characterized by the ratio between storage and loss moduli (G′ and G″) (Terry et al., 2004). Several outstanding fiber spinning approaches with silk feedstocks are based on pH effects, which will be discussed in Section 3.1.

The effects of salt ions and pH on the assembly of silk proteins are not usually decoupled. It is primarily because common pH buffer solutions are always composed of various salt ions. However, organic quaternary amines, such as tetramethylammonium (TMA), may replace sodium and potassium ions to formulate pH buffer solutions and verify the effects of mineral ions on macromolecues, on the solubility of PNIPAM (Bruce et al., 2020), thus promising for silk proteins.

#### 2.2.3 Water content

Along the spinning apparatus, the water, as the solvent for silk proteins, is also actively manipulated, which is gradually reduced, perhaps by the active reabsorption of the epithelium and the evaporation to air after exhibiting the spinneret (Magoshi et al., 1996; Vollrath and Knight, 2001). The removal of water slows down the linear velocity of the spinning dope, which is beneficial for the manipulation of other solvent cues by diffusion, as well as is necessary for the formation of solid and compact structures (Foo et al., 2006). The role of water content or water removal has been recognized as a fundamental mechanism for conformational transition (Hu et al., 2008; Mo et al., 2009; Yazawa et al., 2016; Nishimura et al., 2018). Also, water molecules are a plasticizer to manipulate the flexibility of the polypeptide chains of silk proteins and determine the mechanical performance of silks (Hu et al., 2007; Lawrence et al., 2010; Yazawa et al., 2016; Nishimura et al., 2018).

The intricate control of water content in the spinning dope is related to the assembly of silk proteins in silk spinning (Vollrath and Knight, 2001; Jin and Kaplan, 2003). In particular, the polyethylene oxide (PEO) solutions were used to remove water from silk fibroin solutions, leading to globular-like structures in 0.8–15 µm diameter that derives from the coalescence of micellar-like nanostructures (100–200 nm in diameter) and forms fibrillar structures under shear forces (Jin and Kaplan, 2003). On the basis of the principle of water removal, osmotic stress of poly(ethylene glycol) (PEG) solutions were used to induce the conformational transition of silk proteins (Sohn et al., 2004) and eventually led to an artificial wet-spinning approach with the assistance of organic solvents (Sohn and Gido, 2009).

## 3 Bioinspired biofabrication

The silk spinning-inspired fabrications may exhibit substantial benefits in manufacturing sustainability and biomedical therapeutics compared to industrial polymer fabrication. In addition to recapitulating the extrusion-based fiber spinning, there are other ways to process silk proteins into a variety of valuable structures and materials, such as photocrosslinking (Kim et al., 2018; Mu et al., 2020b; Mu et al., 2022b; Xie et al., 2023), salt leaching and lyophilization (Kim et al., 2005; Tozzi et al., 2018), gel spinning (Lovett et al., 2008), spin coating (Jiang et al., 2007; Bucciarelli et al., 2018; Chen J. et al., 2019), thermomoulding (Guo et al., 2020b), lithography (Kim et al., 2014; Jiang et al., 2018; Zhou et al., 2018), and others (Rockwood et al., 2011; Li et al., 2022). This section discusses two approaches that primarily rely on the recapitulation of the native solvent cues along the spinning apparatus, including fiber spinning via an aqueous acidic bath and 3D printing via an aqueous salt bath.

### 3.1 Fiber spinning

**FIGURE 4:**
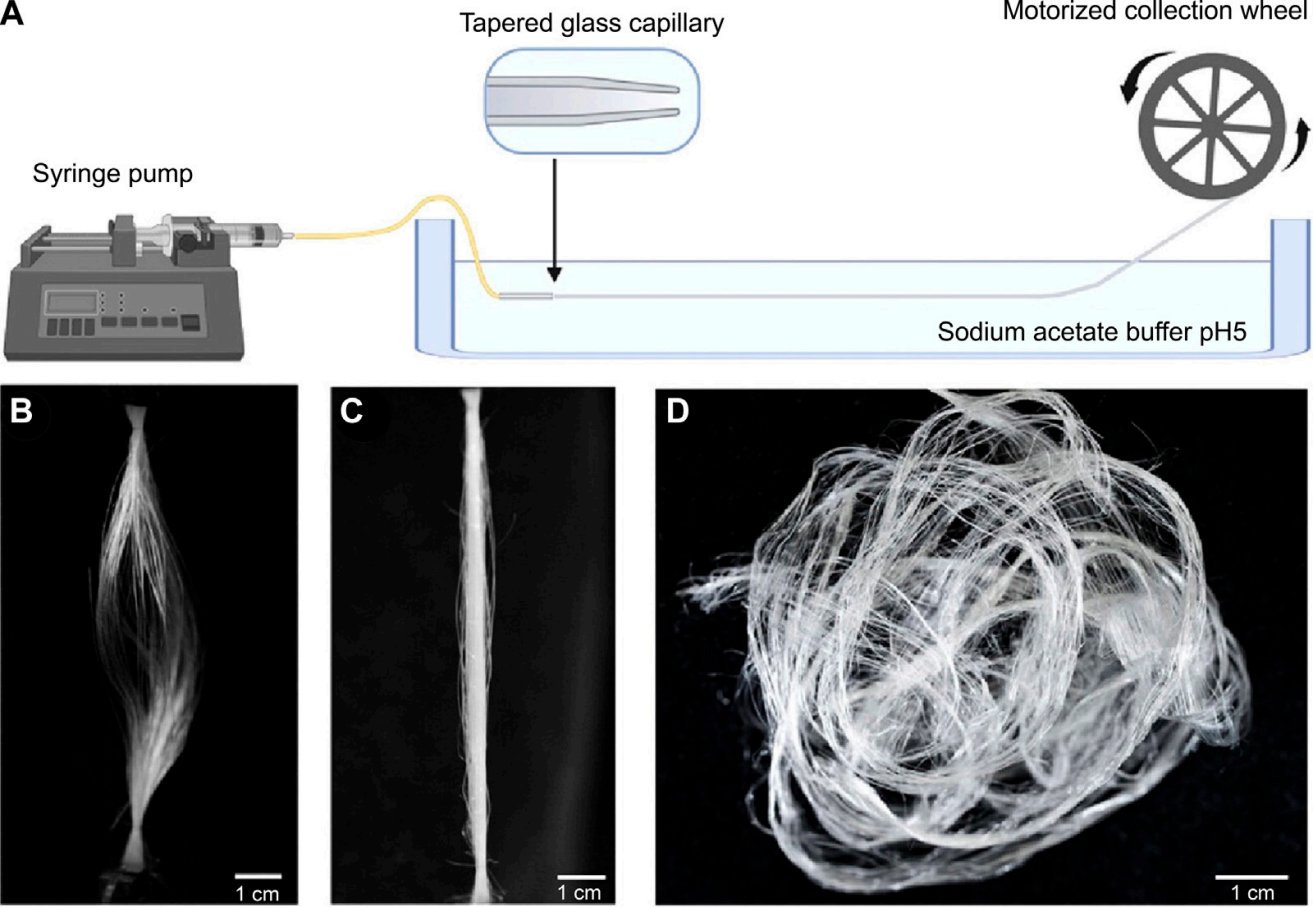
Schematics of artificial fiber spinning with recombinant spider silks. **(A)** Instrument setup. **(B,C)** Slack and straightened status of artificially spun spider silk fibers. **(D)** Artificial silks demounted from the collection wheel. Reproduced with permission from the American Chemical Society (Rising and Harrington, 2023).

Most artificial fabrication with silk protein feedstocks, including fiber spinning and 3D printing, rely on organic solvents, including methanol and isopropanol (Koeppel and Holland, 2017), which aggregate silk proteins in a manner largely different from the native mechanisms and lead to the non-native organization of silk proteins. A whole-aqueous spinning process for artificial fiber spinning has been devised based on the mechanism of pH-mediated assembly (Askarieh et al., 2010; Andersson et al., 2014) with an aqueous bath (500 mM Na-acetate and 200 mM NaCl pH 5.0) and the feedstocks of monolithic recombinant spider silk proteins. It exhibits a toughness of around 45 MJ/m^3^ (Andersson et al., 2017) (Figure 4). When the pH of the bath is below around 3.0 or above 7.0, the extruded silk proteins fail to form continuous filaments. The acidic bath with pH 5.5 also leads to a significant shift toward quaternary structure and β-sheet conformations (Andersson et al., 2017). In another study using the same buffer, the toughness of artificial silk fibers is improved to 74 ± 40 MJ/m^3^ (Schmuck et al., 2021). Furthermore, the artificial spinning of rationally designed spider silk proteins in another acidic bath (750 mM acetate buffer, 200 mM NaCl, pH 5.0) led to the toughness of 146 and 125 MJ/m^3^ (Arndt et al., 2022), which is comparable to 136 MJ/m^3^ of *Argiope argentata* dragline silks (Blackledge and Hayashi, 2006). The rational design is to replace alanine at certain positions with isoleucine, which is claimed to enable the high-yield production of recombinant proteins in prokaryotic hosts as well as enhances the propensity to form β-strands and β-sheets (Johansson et al., 2010).

### 3.2 3D printing

Spiders and silkworms fabricate 3D structures, such as orb webs and cocoons. Thus, silk spinning seems to present a natural version of extrusion-based 3D printing/additive manufacturing. 3D printing based on digital design may provide a range of manufacturing benefits compared to conventional subtractive manufacturing (Heinrich et al., 2019; Zhang et al., 2021). Notably, 3D printing is advantageous in the fabrication of mold-free, digitally designed, patient-specific scaffold with considerable turnaround time and anatomic accuracy that is promising in the treatment of a range of tissue defects. Silk spinning has inspired a range of 3D printing approaches that may use concentrated electrolytes (Lewis, 2006), organic solvents (Ghosh et al., 2008; Jose et al., 2015), and structural additives (Zheng et al., 2018). These outstanding studies have been extensively examined in prior publications (Guo et al., 2020a; Mu et al., 2020a; Agostinacchio et al., 2021; Mu et al., 2021; Chakraborty et al., 2022).

**FIGURE 5:**
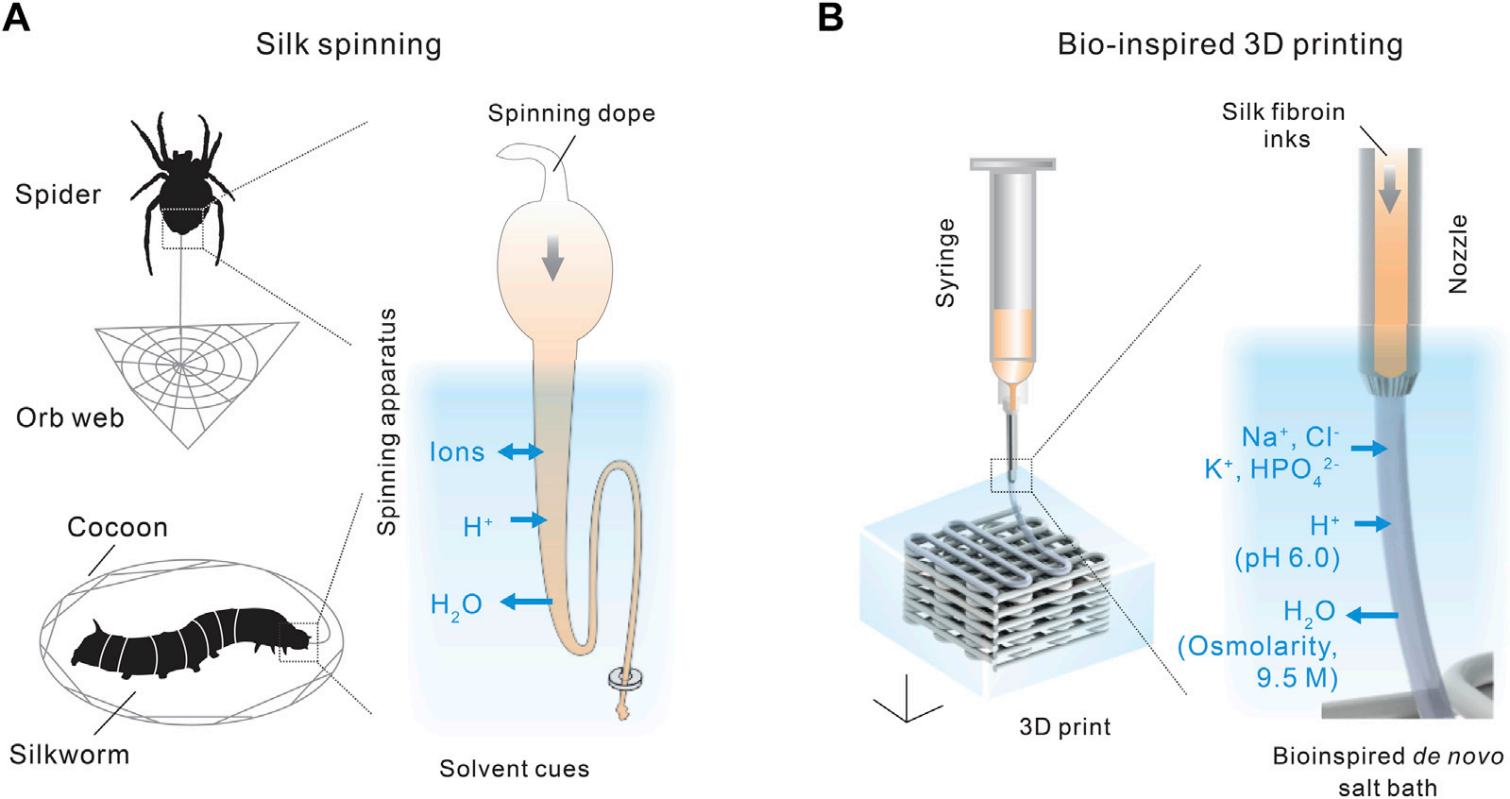
**(A,B)** Comparison between silk spinning and bio-inspired 3D printing. A *de novo* salt bath (4 M NaCl, 0.5 M K_2_HPO_4_, pH 6.0) is devised to mimic the native solvent environment along the spinning apparatus. Reproduced with permission from John Wiley and Sons (Mu et al., 2020c).

**FIGURE 6:**
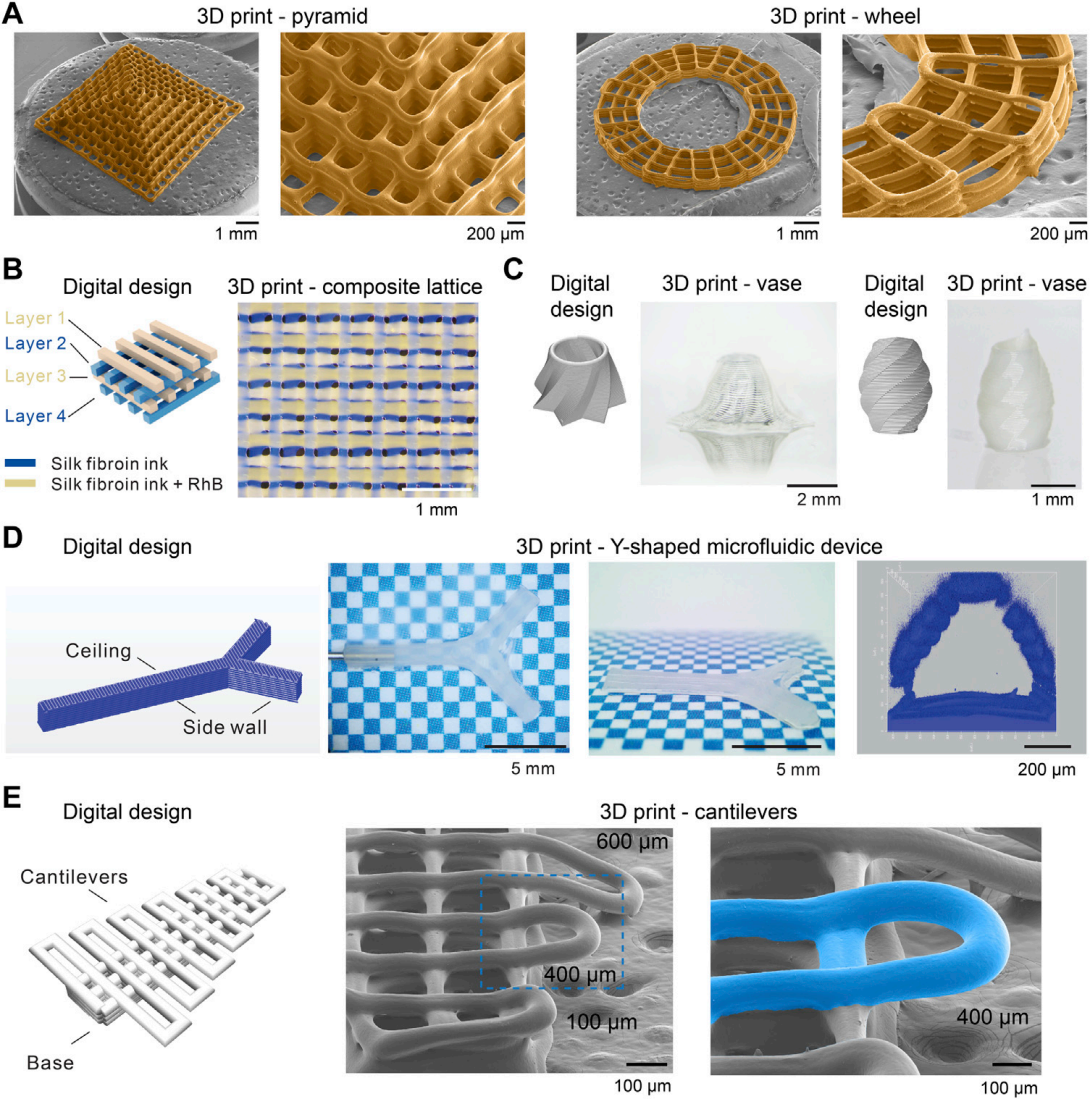
Morphological characterizations of 3D printed silk fibroin structures. **(A)** Scanning electron microscope (SEM) images of 3D-printed silk fibroin pyramid- and wheel-like structures. **(B)** A four-layer 3D printed lattice structure composed of two kinds of inks. **(C)** 3D-printed vase-like silk fibroin structures. **(D)** 3D-printed silk fibroin microfluidic device. **(E)** 3D-printed silk fibroin cantilevers. Reproduced with permission from John Wiley and Sons and under CC-BY (Mu et al., 2020c; Mu et al., 2022a).

Recently, we demonstrated a *de novo* aqueous salt bath for 3D printing with monolithic silk fibroin inks (Mu et al., 2020c; Mu et al., 2022a), which may represent an important step toward silk spinning-inspired biofabrication (Figures 5, 6). The most important technical traits of this 3D printing method include the whole-aqueous and ambient processing conditions (the absence of heating and organic solvents), the monolithic proteinaceous composition of the ink (the elimination of non-protein additives), exceptional printability, and, importantly, a mechanism different from temperature-induced, enzymatic, and ionic crosslinking for the 3D printing with collagen, fibrin, gelatin, and alginate. The synergy of all technical traits is critical to fulfilling the promise of silk spinning-inspired biofabrication in sustainable polymer fabrication and various biomedical applications.

The composition of the salt bath is primarily inspired by the three solvent cues along the spinning apparatus, including ions, pH, and water content, as discussed in Section 2.2. The aqueous salt bath contains 4 M NaCl and 0.5 M K_2_HPO_4_, which brings the K^+^ and HPO_4_^2−^ to the extruded silk protein inks by diffusion. The high salt concentration also leads to a high osmolarity that helps removes water from the extruded silk inks. In addition, the salt bath is slightly acidic, around pH 6.0, which reduces the electrostatic repulsive forces and facilitates the interaction and assembly of silk protein molecules. In the absence of crosslinking chemicals and heating (Gantenbein et al., 2018; Lee et al., 2019), the mechanical performance, especially tensile strength and toughness, of the 3D-printed silk structures is comparable with or superior to most biopolymers (Mu et al., 2020c).

Furthermore, the 3D printing approach exhibited much-improved printability and fidelity compared to other 3D printing with silk protein inks (Jose et al., 2015; Schacht et al., 2015) (Figure 6). 3D-printed silk fibroin pyramid and wheel, imaged by scanning electron microscope (SEM), demonstrated the resolution of filaments around 100 µm and the well-organized connection between filaments and layers. This approach also allows multi-material printing, i.e., the use of two kinds of inks and the construction of a 4-layer composite lattice (Figure 6B). The bioinspired 3D printing approach can print vase-like structures and a Y-shaped perfusable microfluidic device, which involves vertical and high aspect ratio structures (Figures 6C, D). Also, this result indicates the feasibility of printing a functional device, which may be beneficial to speed up the turnaround from the design to the product. In the previous work (Mu et al., 2020c), we also demonstrated that the aspect ratio of the 3D-printed overhanging silk fibroin filament is up to 375, which is higher than other reports, including ∼20 (electrolytes) (Smay et al., 2002), ∼33 (Carbomer) (Chen Z. et al., 2019), and ∼1 (silk fibroin) (Dickerson et al., 2017). The overhanging filament is only supported by the two ends and tends to sag and thus has been suggested to be a criterion for assessing printability (Ribeiro A. et al., 2017). In addition to overhanging filaments, we demonstrated the 3D-printed silk fibroin cantilevers (Mu et al., 2022a) (Figure 6E). A range of cantilevers is printed on top of a base and is supported by only one end, thus more challenging to print than overhanging filaments. The 3D-printed cantilever remains straight without sagging when the span length is around 400 µm and below. The cantilever-like structures seem a valuable alternative to the overhanging filaments for the assessment of the printability and the optimization of the ink composition and printing conditions.

The 3D printability is related to the dynamics of the sol-gel transition of silk fibroin inks. The sol-gel transition should be fast enough to maintain the filamentary morphology of the extruded silk inks, while a too-fast transition may lead to inferior bonding between layers and the clog of dispensing needles. The sol-gel transition can be controlled by the concentration and composition of salt ions. For example, 5M K_2_HPO_4_ bath will lead to a more rapid change of G’ than the bath of 4 M NaCl and 0.5 M K_2_HPO_4_, thus prone to clog the dispensing needle and compromising the 3D printability (Mu et al., 2020c). The cutting off of the extruded silk fibroin filaments is controlled by air pressure.

## 4 Silk spinning for systems sustainability

**FIGURE 7:**
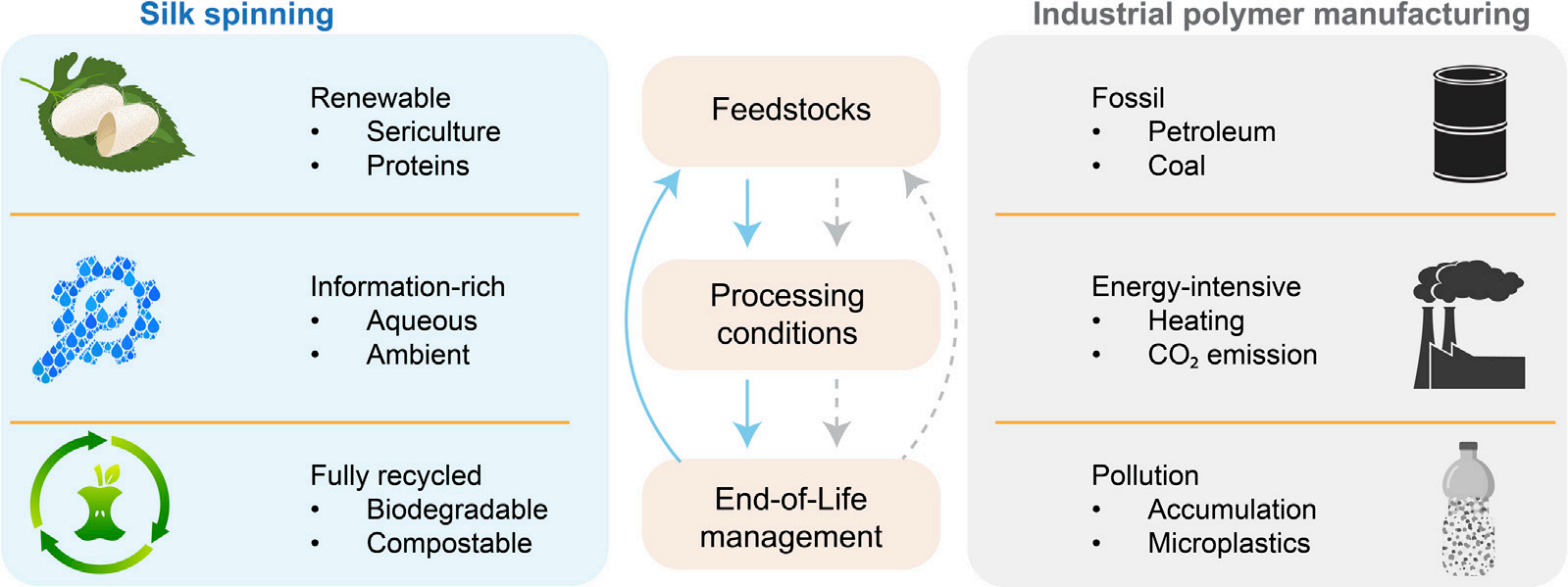
Comparison between silk spinning and industrial polymer manufacturing. Silk spinning may inspire a holistic approach to addressing sustainability challenges in polymer fabrication.

The modern industry of polymer manufacturing largely relies on non-renewable fossil resources and energy-intensive thermal processing (Baird and Collias, 2014; Cabernard et al., 2022), and results in environmental pollution of greenhouse gases (Posen et al., 2017) and (micro)plastics (MacLeod et al., 2021; Vethaak and Legler, 2021) (Figure 7). In addition, global polymeric production is estimated to double by 2045 (Bergmann et al., 2022), thus escalating the sustainability challenges. Notably, most efforts to keep polymer manufacturing sustainable are based on a reductionist approach, which focuses on the improvement of isolated, individual parts yet still relies on other non-sustainable ones.

To keep the sustainability of our society in the coming decades is a multidimensional and nested complex challenge (Anastas, 2019). Thus, systems thinking and approaches become increasingly important in tackling the emerging sustainability challenges in polymer manufacturing (Zimmerman et al., 2020). Systems thinking is characterized by innovative designs at the molecular level for the whole life cycle, circular processes, and an expanded definition of performance with environmental and energy considerations (Zimmerman et al., 2020).

To this end, silk spinning may offer a biological source of inspiration to devise a holistic, sustainable approach to polymer fabrication (Vollrath and Porter, 2009; Tao et al., 2012; Mu et al., 2020a; Li et al., 2022; Rising and Harrington, 2023) (Figure 7). The molecular design of silk proteins is central to the sustainability of silk spinning. Silk proteins are composed of amino acids and can be harvested from sericulture and host animals, which are renewable. Silk proteins can be directed to assemble into hierarchical structures under ambient and aqueous conditions without intense energy input (high temperature and pressure) and organic solvents (Holland et al., 2012; Sparkes and Holland, 2019). The complex information coded in the assembly of silk proteins is believed to reduce the energy input. In contrast, the fabrication of high-performance polymer products often requires high temperatures and pressure. The end-of-life management of silk proteins is convenient, as silk proteins are degradable, compostable and even edible, leading to no environmental accumulation. The *in vivo* and *in vitro* degradation of silk proteins has been scrutinized (Horan et al., 2005; Wang et al., 2008). The degradation byproduct of silk proteins is likely short peptides and amino acids, which could be used in making feedstocks, thus contributing to a circular process. The significant potential of silk spinning in sustainable polymer fabrication highlights the importance of investing in fundamental research on molecular mechanisms and exploring bioinspired advanced fabrication approaches.

## 5 Conclusion

In summary, this article briefly reviews some important mechanisms of silk spinning and bio-inspired biofabrication techniques. The tiny creatures, such as spiders and silkworms, provided sophisticated molecular mechanisms for devising bioinspired polymer fabrication with possibly significant impact on our society. In particular, the monolithic proteinaceous composition and ambient and aqueous processing conditions are highly desired for a holistic approach to addressing emerging challenges in healthcare and sustainability. In addition, the future of silk spinning-inspired biofabrication for biomedical applications seems rosy, especially for hard tissue regeneration (Yan et al., 2012; Melke et al., 2016; Ribeiro V. P. et al., 2017; Cheng et al., 2018; Fitzpatrick et al., 2021), bioelectronics, vascular grafts (Lovett et al., 2010; Bosio et al., 2017; Tanaka et al., 2021), and nerve conduits (Madduri et al., 2010; Alessandrino et al., 2019; Carvalho et al., 2021), and has drawn substantial attention from academia and industry globally (Kundu et al., 2013; Holland et al., 2019). A major hurdle for devising silk-spinning-inspired biofabrication is the incomplete understanding of the exact molecular mechanisms and native solvent cues. We envision that future advances in the field of silk spinning-inspired biofabrication will be driven by collaborations between multiple disciplines and the critical need for promoting sustainability and devising high-value biomedical tools.
